# Super-Resolution Reconstruction of Cell Pseudo-Color Image Based on Raman Technology

**DOI:** 10.3390/s19194076

**Published:** 2019-09-20

**Authors:** Yifan Yang, Ming Zhu, Yuqing Wang, Hang Yang, Yanfeng Wu, Bei Li

**Affiliations:** 1 Changchun Institute of Optics, Fine Mechanics and Physics, Chinese Academy of Sciences, Changchun 130033, China; yangyifan_by@sina.com (Y.Y.); zhu_mingca@163.com (M.Z.); yanghang@ciomp.ac.cn (H.Y.); bei.li@ciomp.ac.cn (B.L.); 2 University of Chinese Academy of Sciences, Beijing 100049, China

**Keywords:** Raman spectroscopy, imaging, peak, deep neural network, pseudo-color

## Abstract

Raman spectroscopy visualization is a challenging task due to the interference of complex background noise and the number of selected measurement points. In this paper, a super-resolution image reconstruction algorithm for Raman spectroscopy is studied to convert raw Raman data into pseudo-color super-resolution imaging. Firstly, the Raman spectrum data of a single measurement point is measured multiple times to calculate the mean value to remove the random background noise, and innovatively introduce the Retinex algorithm and the median filtering algorithm which improve the signal-to-noise ratio. The novel method of using deep neural network performs a super-resolution reconstruction operation on the gray image. An adaptive guided filter that automatically adjusts the filter radius and penalty factor is proposed to highlight the contour of the cell, and the super-resolution reconstruction of the pseudo-color image of the Raman spectrum is realized. The average signal-to-noise ratio of the reconstructed pseudo-color image sub-band reaches 14.29 db, and the average value of information entropy reaches 4.30 db. The results show that the Raman-based cell pseudo-color image super-resolution reconstruction algorithm is an effective tool to effectively remove noise and high-resolution visualization. The contrast experiments show that the pseudo-color image Kullback–Leiber (KL) entropy of the color image obtained by the method is small, the boundary is obvious, and the noise is small, which provide technical support for the development of sophisticated single-cell imaging Raman spectroscopy instruments.

## 1. Introduction

The Raman spectrum is a scattering spectrum obtained by the Raman scattering effect. Based on strong molecular specificity [1], Raman spectroscopy has the advantage of non-invasive, high specificity, and high sensitivity [2,3]. It has a wide range of applications in geology, medicine, archaeology, and chemistry [4,5,6,7,8,9,10,11]. For example, Li et al. [12] have studied Sudan Red I in duck feed by analyzing the R, G, B three-color channel of the Raman spectral pseudo-color images and the Raman pseudo-color image binarization. Chao et al. [13] developed a Raman spectral imaging system for food safety and quality assessment, which was capable of high-spectrum Raman imaging. However, the Raman spectroscopy two-dimensional images contained some noise. Qin et al. [14] developed a line-scan Raman spectroscopy imaging platform that could evaluate food safety and internal quality. The platform increased the image resolution by increasing the number of scanning points, but if the number of scanning points was small and the object size was close to the scanning limit, which was 0.07 mm, the imaging would be blurred. Anna et al. [15] studied the Raman imaging of brain tumors by further processing the obtained wavelength information and combining the pseudo-color information. It was found that there were significant metabolic differences between high-grade medulloblastoma and normal brain tissue. Lohumi et al. [16] used Raman imaging to detect the content of dopants in paprika, by analyzing the univariate, bivariate, and multivariate of the Raman spectra of the substances, the method has effectively reduced the errors caused by the background noise interference. Hauke et al. [17] presented a novel approach to in situ studies for the sintering process of silicate ceramics by hyperspectral Raman imaging, which demonstrated the power of hyperspectral Raman imaging for in situ studies of the mechanism(s) of solid-solid or solid-melt reactions at high-temperature with a micrometer-scale resolution. Wada et al. [18] used the Raman technique and studied the Raman images of carriers (positive polarons) at the channel of an ionic liquid-gated transistor (ILGT) fabricated with region-regular poly (3-hexylthiophene) (P3HT) with excitation at 785 vnm. Kopec et al. [19] combined high-resolution pseudo-color Raman imaging to determine the biochemical composition and mechanical topography around blood vessels in the tumor mass of human breast tissue and found that there are significant alterations of the chemical composition and architecture around the blood vessel compared to the normal breast tissue.

In Raman imaging, the main hot spot is to make nanomaterials enter cells through endocytosis and exocytosis and to study the properties of nanomaterials or the metabolic mechanism of cells by Raman spectroscopy scanning of cells [20]. High-resolution images could be generated by coherent anti-Stokes Raman spectroscopy (CARS), which makes it easier to identify subcellular features [21]; and stimulated Raman scattering enhances the excitation efficiency, increasing the image acquisition speed by 1000 times. For hyperspectral Raman imaging, principal component analysis (PCA), and vertex component analysis (VCA) are commonly used to analyze complex raw data [22,23]. Since the biological function of cells is affected by biologically active small molecules, in recent years, the means using Raman tags, such as deuterium, nitrile, and alkyne, have been proposed to detect small molecules in biological samples [24]. Raman cell imaging technology is developing rapidly, but there are a few studies on the algorithm of Raman two-dimensional pseudo-color imaging. This paper establishes a set of algorithms from hyperspectral data to pseudo-color imaging. It is worth emphasizing that most studies on Raman spectroscopy have segmented features. It is also worth noting that the above segmentation refers to the in-depth study of Raman data at specific steps. However, for the holistic process of processing Raman data from single, discontinuous waveform data into visualized pseudo-color imaging, there are a few studies. 

The Raman spectral resolution is between 200 and 500 nm, while the resolution of the digital optical microscope is on the submicron scale (about 0.2 µm). The two are not comparable in accuracy, and the digital optical microscope image has more detailed information. However, the digital optical microscope obtains an image of the surface of the substance, which is close to the grayscale image and has few color information. Therefore, digital optical microscopy is difficult to detect the substances or structures inside cells. However, for some agricultural products with outer skin wrap and nanocarriers inside the cell, although ultrasound, X-ray, and magnetic resonance imaging (MRI) have been used to evaluate the internal characteristics of the test object, the information obtained is limited due to the lack of compound-specific information [25]. Therefore, there is a need for a technique that could detect both the internal components of the object and the spatial resolution, while Raman spectroscopy is meeting the requirements. How to generate a clear, accurate, and contrast-like image in the Raman data which is accompanied by huge noise becomes very important.

**Figure 1:**
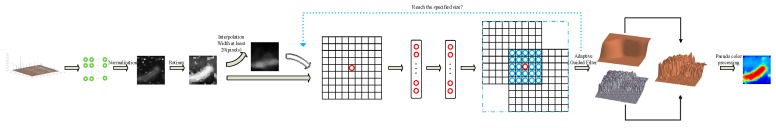
An illustration of the proposed methods.

This paper proposes a unified algorithm shown in Figure 1 from Raman data to pseudo-color images. For the obtained Raman signal with certain noise, the average value is obtained by multiple measurements, and the peak information is directly extracted, arranged into a gray image according to the measurement point order. The image enhancement processing is performed using the Retinex theory and the image denoising is performed by median filtering. The bicubic interpolation method and the pre-trained Super-Resolution Convolutional Neural Network (SRCNN) network are used for super-resolution processing. Finally, the adaptive guided filtering method is used to smooth the image, which innovatively uses the linear fitting method to select the radius and regularization coefficient in guided filtering adaptively. After obtaining a Raman spectral grayscale image, the Jet sequence in Matlab is used to pseudo-color process the image. Thus, pseudo-color imaging of the Raman spectrum is achieved.

## 2. Materials and Methods

**Figure 2:**
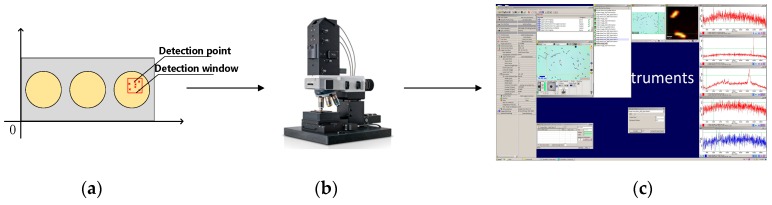
Acquisition of spectra by a Raman spectrometer. (**a**) The schematic of the determination of the detection points. (**b**) Spectral data acquisition. (**c**) Spectral analysis.

In this experiment, the cells immobilized with alcohol in glass sections (including Escherichia coli (dh5a strain), yeast cells (Yeast), and human colon cancer cells (the hct116 cell line) were obtained from Hooke Instruments, Changchun, China and used as experimental samples. The three kinds of cells have certain representativeness: Escherichia coli cells are small, yeast cells have the characteristics of aggregation, and human colon cancer cells have a representatively large volume. The test samples were placed under a microscope at ambient room temperature for observation. In this paper, the Witec Alpha 300R instrument is used for collecting Raman spectra. The instrument consists of a digital controller, a laser spectrometer, and a charge coupled device camera. The measured Raman spectral light intensity is saved using the Control FIVE software provided by Witec Instruments. Digital optical microscopy imaging and Raman spectroscopy pseudo-color imaging shown in Figure 2 were performed by the Control FIVE software. The device is used when the CCD temperature is lowered to −60 °C for maximum sensitivity. All of the images included a field of view equal or less than 20 × 20 µm. In this paper, the collected samples were scanned using a 534 nm laser. All of the samples were computed at 3–5 cm^−1^ resolution across the spectral range of 155–3926 cm^−1^. The integration time ranged from 2 s to 10 s depending on the sample. The laser power ranged from 1.5 mw to 11 mw depending on the sample. Single-point spectral scanning was used to set the test point <= 400 points, and a total of two scans were obtained for each spectrum.

## 3. Related Work

### 3.1. Retinex Image Enhancement Technology

**Figure 3:**
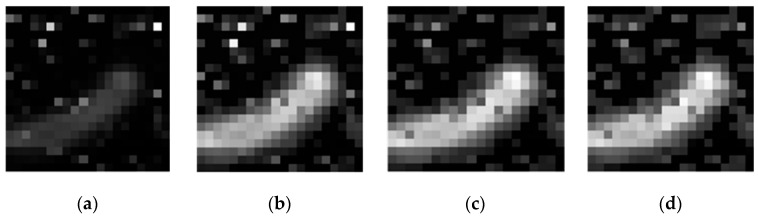
N points represent the symmetrical points near the peak. The four images are, respectively, the result of extracting the N values around the peaks. In Figure (**a**), N = 1. In Figure (**b**), N = 4. In Figure (**c**), N = 8. In Figure (**d**), N = 12.

Extracting useful information in a short period of time is a very important task because the data measured by Witec instruments is superimposed on machine noise, fluorescence noise, and phosphorescence noise. The averaging of the measurement noise could effectively suppress machine noise. For fluorescence noise and phosphorescence noise, it is difficult to form a unified method to remove it due to the instability of ambient temperature and substrate enhancement properties. Specific spectral denoising process is another area of research for Raman spectroscopy and is not be described here. The research in this paper is to carry out clear imaging processing of Raman spectroscopy under the premise of minimal pretreatment, which provides a guarantee for Raman spectroscopy in other fields. This paper does not denoise the Raman spectral data from the perspective of data processing, but innovatively denoises the Raman spectral data in the field of image processing. It is also tested shown in Figure 3 that the symmetrical N (N = 4, 8, 12) points near the peak are averaged, and the image contrast is found to be large, but the noise is also increased. It is observed that a “void” phenomenon occurs inside the cell. Filtering these salt and pepper noises in the case of strong contrast increases the difficulty of the next work.

Therefore, the initial image is obtained by using the method of multi-measurement peak averaging. Therefore, it is necessary first to improve the contrast of the image, and use the Retinex theory to improve the contrast processing of the image [26].

**Figure 4:**
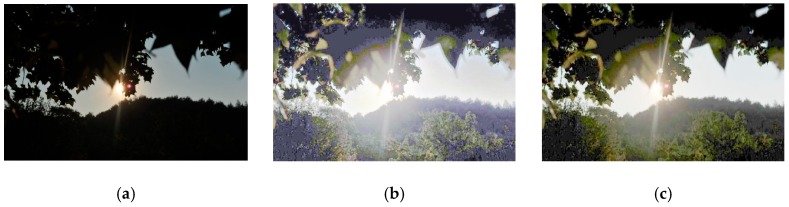
Comparison of the Retinex algorithm and the histogram equalization algorithm in image enhancement. Figure (**a**) is the original image. Figure (**b**) is enhanced by the histogram equalization algorithm. Figure (**c**) is enhanced by the Retinex algorithm.

The effect of the Retinex algorithm is shown below. In the original picture, the overall brightness of the image is low, and the details in the dark area cannot be seen clearly; the histogram equalization and the Retinex algorithm both enhance the contrast of the image, enhance the details of the dark area, and observe the Figure 4b,c the histogram equalized image has halo and artifacts, and the color distortion is more serious. The Retinex algorithm performs better in these aspects. Therefore, the Retinex algorithm is used to perform contrast stretching on the obtained Raman gray image. Figure 4 shows the superiority of the Retinex algorithm for enhanced images.

Retinex theory believes that the color of an object is only related to the ability of the object to reflect long, medium and short waves, and is independent of the intensity of the incident light, the non-uniformity of the light, and the absolute intensity of the reflected light. Therefore, in the Retinex image enhancement algorithm, the image to be enhanced is decomposed into two parts, an incident component and a reflection component, and the difference in brightness between the pixels (i.e., the difference in gray value) is compared to obtain an incident component. Then, the reflection component is obtained by stretching or the like operation. Finally, the effect of image enhancement is achieved. When calculating the relative shading relationship, assume that the size of the image is m×n, and assume the light and dark values of each pixel are the same at the beginning, given by:

(1) $$L_{\mathrm{uminance}}\left[i\right]\left[j\right]=C_{\mathrm{onstant}}\;1\leq i\leq m,1\leq j\leq n$$

Here $L_{\mathrm{uminance}}\left[i\right]\left[j\right]$ indicates that the gray value at the position of the image (i,j) takes a log function, and $C_{\mathrm{onstant}}$ is a certain matrix, i.e., $C_{\mathrm{onstant}}{=\log}_{10}\left(P\right)$.

First calculate the relative light and dark relationship between any two pixels with a distance $h=\frac{m}{2}$, given by

(2) $$L_{\mathrm{uminance}}\left[i\right]\left[j\right]=L_{\mathrm{uminance}}\left[i\right]\left[j\right]+\log_{10}\left(\frac{d\left[i+h\right]\left[j\right]}{d\left[i\right]\left[j\right]}\right)$$

then compare with the $C_{\mathrm{onstant}}$ value, as shown in the following

(3) $$L_{\mathrm{uminance}}\left[i\right]\left[j\right]=\{\begin{array}{cc} C_{\mathrm{onstant}}\left[i\right]\left[j\right] & L_{\mathrm{uminance}}\left[i\right]\left[j\right]>C_{\mathrm{onstant}}\left[i\right]\left[j\right]\\ L_{\mathrm{uminance}}\left[i\right]\left[j\right] & L_{\mathrm{uminance}}\left[i\right]\left[j\right]<C_{\mathrm{onstant}}\left[i\right]\left[j\right] \end{array}$$

then calculate the relative brightness relationship between any two pixels in the vertical direction with distance $\vartheta =\frac{n}{2}$, i.e.,

(4) $$L_{\mathrm{uminance}}\left[i\right]\left[j\right]=L_{\mathrm{uminance}}\left[i\right]\left[j\right]+\log_{10}\left(\frac{d\left[i\right]\left[j+\vartheta \right]}{d\left[i\right]\left[j\right]}\right)$$

Equation (4) is also compared with $C_{\mathrm{onstant}}$, the formula is the same as Equation (3). After calculating the relative shading relationship value of each pixel, the distance in the horizontal direction is reduced to $h=\frac{m}{4}$, the distance in the vertical direction is reduced to $\vartheta =\frac{n}{4}$, and the iterative calculation is repeated until the distance between the horizontal and vertical directions is 1. Finally, according to the gray maximum value and the gray minimum value in the processed image, uniform stretching processing is performed [27].

**Figure 5:**
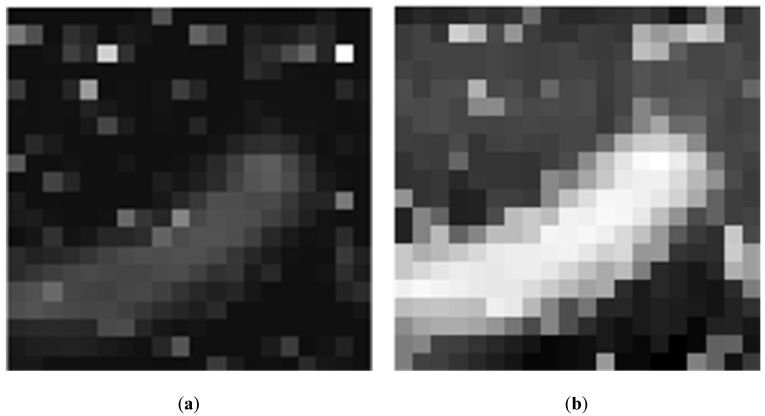
(**a**) represents the original grayscale cell image after extracting the peak. (**b**) represents the Figure which has been enhanced by the Retinex algorithm.

The image is processed and the result is shown in Figure 5.

### 3.2. Image Super-Resolution Reconstruction

The traditional super-resolution methods are bilinear interpolation, bicubic interpolation, sparse coding-based methods, anchored neighborhood regression methods, etc., while deep learning methods are superior to these algorithms [28].

**Figure 6:**
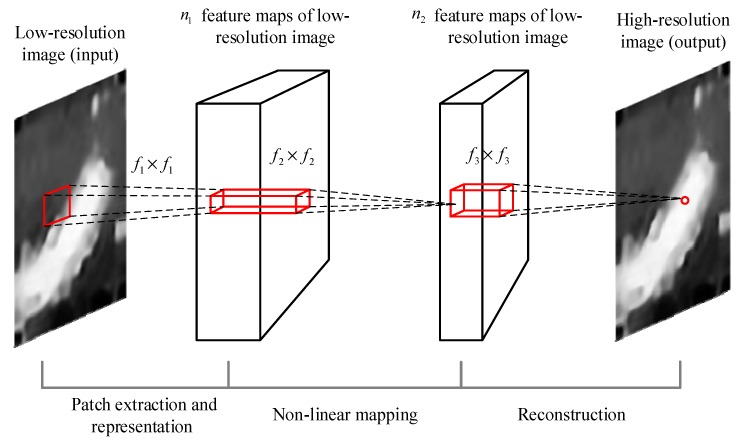
The Super-Resolution Convolutional Neural Network deep neural network.

**Figure 7:**
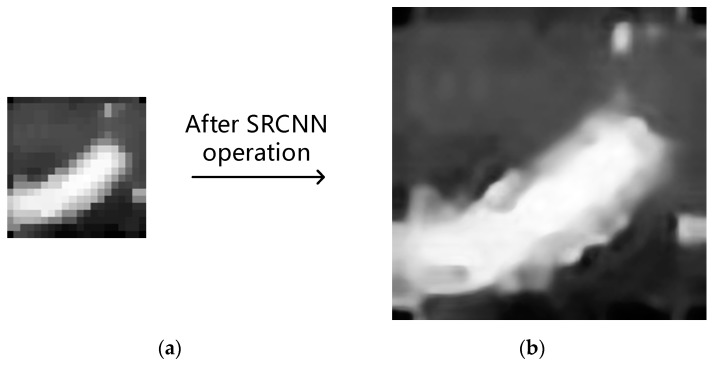
(**a**) Before the SRCNN operation. (**b**) After the SRCNN operation.

It could be seen from [28] that the Super-Resolution Convolutional Neural Network (hereinafter referred to as SRCNN) achieves a better super-resolution effect. The test selects the Set 14 image data set for testing, and the SRCNN is superior in most of the evaluation indicators. Therefore, this paper uses SRCNN network for super-resolution processing. SRCNN is a three-layer deep neural network, as shown in Figure 6:

Formally, the first layer is represented as operation $F_{1}$:

(5) $$F_{1}\left(Y\right)=\max\left(0,W_{1}\times Y+B_{1}\right)$$

where $W_{1}$ is a convolution kernel with size 64 × 9 × 9, and the size of $B_{1}$ is 64 × 1. The second layer is represented as operation $F_{2}$:

(6) $$F_{2}\left(Y\right)=\max\left(0,W_{2}\times F_{1}\left(Y\right)+B_{2}\right)$$

where $W_{2}$ contains 32 convolution kernels with size 64 × 5 × 5, and $B_{2}$ size is 32 × 1. The third layer is represented as $F_{3}$ operation:

(7) $$F_{3}=W_{3}\times F_{2}\left(Y\right)+B_{3}$$

where $W_{3}$ is a convolution kernel with size 32 × 5 × 5, and the size of $B_{3}$ is 1 × 1. In the SRCNN network, each neuron selects the Rectified Liner Uints (ReLU) function as the activation function and uses the symmetric extension method to perform the convolution operation at the image boundary. The network is pre-trained in the ImageNet dataset, and the trained network is directly applied to the low-resolution images in this paper shown in Figure 7. Through two super-resolution deep neural networks, it is found that the network enhances the contour edge of the image, but also enhances some noise point information.

### 3.3. Adaptive Guided Filter

#### 3.3.1. Traditional Guided Filter

**Figure 8:**
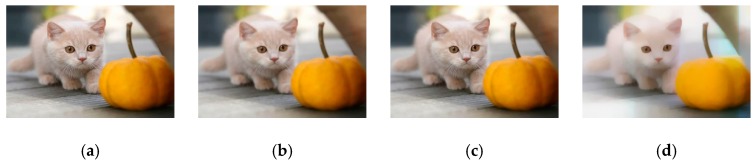
The comparison results of different filters. (**a**) The original image. (**b**) After guided filtering. (**c**) After bilateral filtering. (**d**) After weighted least squares filtering (WLS).

Since the images processed by the SRCNN network are inconsistent in terms of details, the image is processed by filtering. There are three main methods for smoothing traditional image smoothing: weighted least squares filtering [29], bilateral filtering [30], and guided filtering [31]. Weighted least squares filtering needs to calculate the inverse of high-dimensional matrices, which is difficult to implement in practical engineering. and the calculation speed is slow. The bilateral filtering also has the problem of long-running time and it has edge inversion characteristics [32]. The guided filtering considers the intrinsic relationship between the pixels of the image, and the model of the ridgeline plays a better role in smoothing the image. Besides, the algorithm runs faster. Therefore, this paper uses guided filtering to smooth the image. Referring to the [31] paper, the guided filtering parameters are set to the radius r = 2, eps = $0.1^{2}$, and the bilateral filtering parameters are set to the radius r = 2, sigma_s = 2, sigma_r = 0.1. When filtering the image, the R, G, and B color channels are separately filtered. The filtered effect diagram is shown in the Figure below. The Weighted least squares (WLS) filter loses a lot of the image details, such as the texture information and the edge information. The bilateral filtering preserves the texture information better, but it can be seen that there is a white edge phenomenon at the edge. The texture information and the edge information are better preserved, and there is no white edge phenomenon at the edge. Figure 8 shows the effects of guided filtering, bilateral filtering, and WLS filtering. Therefore, the super-resolution image is operated by the guided filter.

The guided filtering algorithm assumes that there is a locally corresponding linear relationship between the guided image I and the output image q. Assuming p is the input image, q is a local linear transformation of the sliding window $\omega_{k}$ centered on pixel k with respect to I, then the following equation:

(8) $$q_{i}=a_{k}I_{i}+b_{k},\forall i\in \omega_{k}$$

By minimizing the following cost function:

(9) $$E\left(a_{k},b_{k}\right)=\sum_{i\in \omega_{k}}\left[{\left(a_{k}I_{i}+b_{k}-p_{i}\right)}^{2}+\varepsilon a_{k}^{2}\right],$$

we solve the coefficients $a_{k}$ and $b_{k}$. In Equation (9), ε is a penalty term coefficient for $a_{k}$. According to the linear ridge regression model, there is

(10) $$a_{k}=\frac{\frac{1}{\left|\omega \right|}\sum_{i\in \omega_{k}}I_{i}p_{i}-\mu_{k}{\bar{p}}_{k}}{\sigma_{k}^{2}+\varepsilon }$$

(11) $$b_{k}={\bar{p}}_{k}-a_{k}\mu_{k}$$

where $\mu_{k}$ and $\sigma_{k}^{2}$ are the mean and variance of p in the sliding window $\omega_{k}$, |ω| is the number of data contained in the sliding window $\omega_{k}$, and ${\bar{p}}_{k}=\frac{1}{\left|\omega \right|}\underset{i\in \omega_{k}}{\sum }p_{i}$ is the mean of the input image p in the sliding window $\omega_{k}$. Since the values obtained by using Equation (8) to cover all overlapping windows at the i position are different, the $q_{i}$ values corresponding to each position are averaged, corresponding to all sliding windows $\omega_{k}$ in the calculated image $\left(a_{k},b_{k}\right)$.

According to the symmetry of the box filter, then $\underset{k|i\in \omega_{k}}{\sum }a_{k}=\underset{k\in \omega_{i}}{\sum }a_{k}$, so eventually rewrite Equation (12) as

(12) $$q_{i}=\frac{1}{\left|\omega \right|}\underset{k|i\in \omega_{k}}{\sum }\left(a_{k}I_{i}+b_{k}\right)$$

(13) $$q_{i}={\bar{a}}_{i}p_{i}+{\bar{b}}_{i}$$

where ${\bar{a}}_{i}=\frac{1}{\left|\omega \right|}\underset{k\in \omega_{i}}{\sum }a_{k},{\bar{b}}_{i}=\frac{1}{\left|\omega \right|}\underset{k\in \omega_{i}}{\sum }b_{k}$. The guided filter has the characteristics of fast speed, edge retention, and no edge inversion, and is essentially an implicit filter. In this paper, the guide image I is the same as the input image p, then $a_{k}$ and $b_{k}$ are simplified to $a_{k}=\frac{\sigma_{k}^{2}}{\left(\sigma_{k}^{2}+\varepsilon \right)},b_{k}=\left(1-a_{k}\right)\mu_{k}$.

#### 3.3.2. Adaptive Guided Filter

This paper innovatively proposes an adaptive guided filtering method. When using traditional guided filtering, it is necessary to manually set the filter radius and regularization coefficient, which makes it difficult to process different cell images. Therefore, this paper proposes a method for adaptively setting the filter radius and regularization coefficient. By the prior image information with its corresponding filter radius and regularization coefficient information, the mapping relationship which also combines with the total Raman spectrum sample points and the sub-band spectrum variance information is fitted to a linear relationship. The final linear expression is obtained by the least-squares method to achieve the optimal guiding filtering effect. Then a moderate result will be obtained in the boundary preservation and image smoothing.

**Figure 9:**
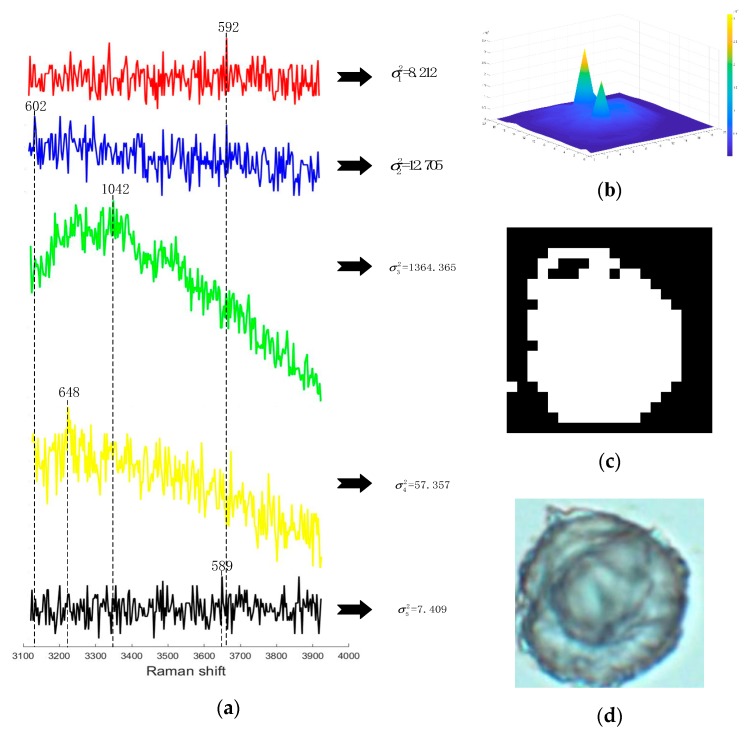
(**a**) Select the original Raman signal in the band. (**b**) Select the band variance depth map. (**c**) Set the binar variance graph after the threshold is 1000. (**d**) Cell image under a digital optical microscope.

After analyzing the variance of the sub-band spectrum, it is observed that when the variance threshold is set to 1000, the difference map which is binarized could better fit the cell boundary, as shown in the above Figure 9c. The reason is that the Raman spectrum intensity changes more dramatically because of the detection of the cell. Therefore, the least-squares fit of r and eps is performed by a large amount of experimental data. The equation of the filter radius is $\left|\omega \right|=a_{1}N_{1}+b_{1}N_{2}+c_{1}\sigma_{k}^{2}+d_{1}$, the equation of eps is $\mathrm{eps}\;=a_{2}N_{1}+b_{2}N_{2}+c_{2}\sigma_{k}^{2}+d_{2}$. Here, $N_{1}$ represents the total number of points scanned, and $N_{2}$ represents the number of the scanning points in the image with the variance of more than 1000, i.e., $N_{2}=\{\text{number of i}|i\in N\;\mathrm{and}\;\sigma_{i}^{2}>1000\}\sigma_{k}^{2}$ represents the intensity variance of a single scan point over the sub-band. The overall fit function is shown below:

(14) $$\left[\begin{array}{c} \left|\omega \right|\\ eps \end{array}\right]=f\left(N_{1},N_{2},\sigma_{k}^{2}\right)=\left[\begin{array}{c} a_{1}\\ a_{2} \end{array}\right]N_{1}+\left[\begin{array}{c} b_{1}\\ b_{2} \end{array}\right]N_{2}+\left[\begin{array}{c} c_{1}\\ c_{2} \end{array}\right]\sigma_{k}^{2}+\left[\begin{array}{c} d_{1}\\ d_{2} \end{array}\right]$$

The loss function is $E\left(A,B,C,D\right)=\overset{n}{\underset{i=1}{\sum }}{\left(Actual-f\left({\left(N_{1}\right)}_{i},{\left(N_{2}\right)}_{i},\sigma_{i}^{2}\right)\right)}^{2}$, here $A=\left[\begin{array}{c} a_{1}\\ a_{2} \end{array}\right]$, $B=\left[\begin{array}{c} b_{1}\\ b_{2} \end{array}\right]$, $C=\left[\begin{array}{c} c_{1}\\ c_{2} \end{array}\right]$,$\;D=\left[\begin{array}{c} d_{1}\\ d_{2} \end{array}\right]$, then the loss is transformed into a matrix form of Ab=Y,

(15) $$a_{i}N_{1}^{\left(1\right)}+b_{i}N_{2}^{\left(1\right)}+c_{i}{\left(\sigma_{k}^{\left(1\right)}\right)}^{2}+d_{i}=y_{i}^{\left(1\right)}$$

(16) $$a_{i}N_{2}^{\left(2\right)}+b_{i}N_{2}^{\left(2\right)}+c_{i}{\left(\sigma_{k}^{\left(2\right)}\right)}^{2}+d_{i}=y_{i}^{\left(2\right)}\vdots$$

⋮

(17) $$a_{i}N_{1}^{\left(n\right)}+b_{i}N_{2}^{\left(n\right)}+c_{i}{\left(\sigma_{k}^{\left(n\right)}\right)}^{2}+d_{i}=y_{i}^{\left(n\right)}$$

where $y\left(N_{1},N_{2},\sigma_{k}^{2};a_{i},b_{i},c_{i},d_{i}\right)=a_{i}N_{1}+b_{i}N_{2}+c_{i}\sigma_{k}^{2}+d_{i}$, i=1, 2.

The matrix is then listed as:

(18) $$\left(\begin{array}{cccc} N_{1}^{\left(1\right)} & N_{2}^{\left(1\right)} & {\left(\sigma_{k}^{\left(1\right)}\right)}^{2} & 1\\ N_{1}^{\left(2\right)} & N_{2}^{\left(2\right)} & {\left(\sigma_{k}^{\left(2\right)}\right)}^{2} & 1\\ \vdots & \vdots & \vdots & \vdots \\ N_{1}^{\left(n\right)} & N_{2}^{\left(n\right)} & {\left(\sigma_{k}^{\left(n\right)}\right)}^{2} & 1 \end{array}\right)\left(\begin{array}{c} a_{i}\\ b_{i}\\ c_{i}\\ d_{i} \end{array}\right)=\left(\begin{array}{c} y_{i}^{\left(1\right)}\\ y_{i}^{\left(2\right)}\\ \vdots \\ y_{i}^{\left(n\right)} \end{array}\right)$$

Solve |ω| and eps by minimizing E(A,B,C,D). Minimizing E(A,B,C,D) is achieved by least squares linear fit $b={\left(A^{T}A\right)}^{-1}A^{T}Y$. Finally, the calculated parameter matrix is $\left[\begin{array}{c} a_{1}\\ a_{2} \end{array}\right]=\left[\begin{array}{c} 0.0113\\ -0.0797 \end{array}\right]$, $\left[\begin{array}{c} b_{1}\\ b_{2} \end{array}\right]=\left[\begin{array}{c} -0.0056\\ 0.1210 \end{array}\right]$, $\left[\begin{array}{c} c_{1}\\ c_{2} \end{array}\right]=\left[\begin{array}{c} 0.0001\\ -0.0001 \end{array}\right]$, $\left[\begin{array}{c} d_{1}\\ d_{2} \end{array}\right]=\left[\begin{array}{c} 3.1181\\ 44.2304 \end{array}\right]$.

In the popular sense, when the number of sampling points is small, the sampled data will have a large noise. At this time, the image quality will be poor, and the image will be blurred, so the size of the filter radius should be reduced. When the variance of the corresponding band of a certain point is large, the overall image area here varies greatly, and r should be reduced, which is consistent with the obtained linear system. Then, the reliability of the obtained linear function is verified from the side.

**Figure 10:**
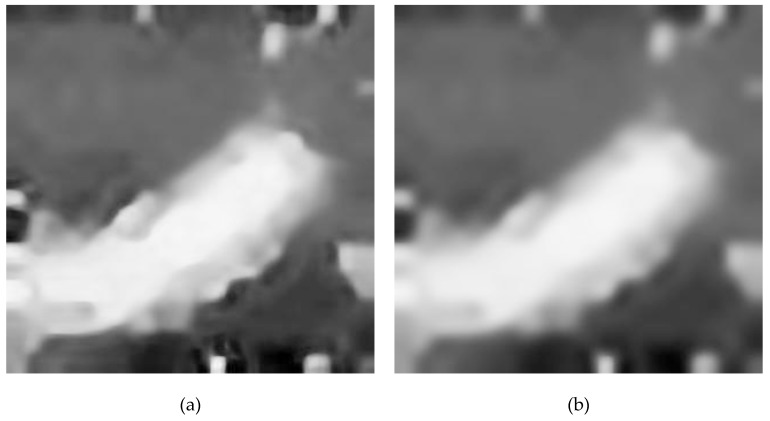
(**a**) The image to be processed. (**b**) After adaptive guided filtering.

By using the adaptive guided filter, the Figure 10b is obtained. The contour of the cells in the image is highlighted, and some of the noise regions are filtered and removed, which is beneficial to the pseudo-color processing of the image in the next step.

### 3.4. Raman Spectral Pseudo-Color Imaging System

**Figure 11:**
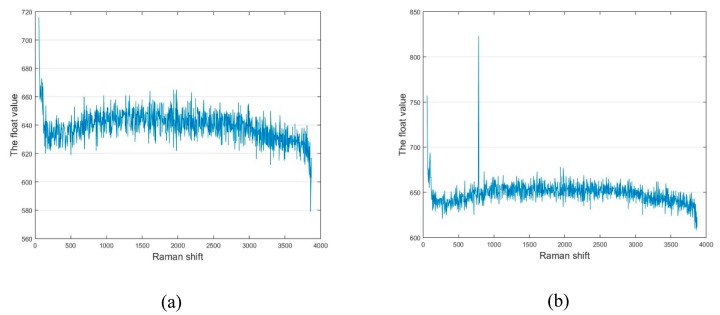
(**a**,**b**) Raman raw measurement data.

Raman data has strong noise and large data volume. Therefore, when performing Raman spectrum pseudo-color imaging, how to extract useful information in a short time and convert it into a high-resolution image is a difficulty. The measured Raman spectrum usually contains noise such as fluorescent background noise, Gaussian noise and shot noise, and the characteristic peak information of the substance to be tested is aliased in the noise. The Figure 11 shows the Raman spectrum information of the measured single observation points.

**Figure 12:**
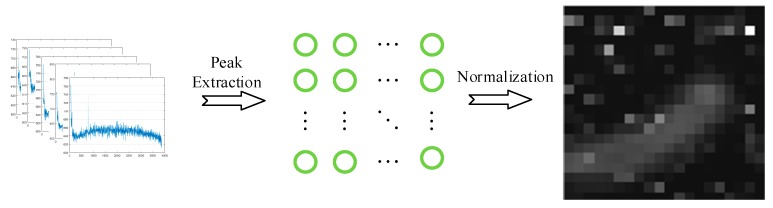
Flow chart of the conversion from Raman spectral data to image.

Raman spectroscopy is the intensity data derived from the energy released by the energy level transition of an electron. Therefore, if the intensity of the obtained amplitude signal is stronger, the possible type of element is larger. According to [2], some of the smaller spectral information is likely to be noise mixing, and the highest peak information selected can better preserve the intensity information. Besides, according to [33], Raman spectral data measured at the same time show similarity, so the peak ratio can better reflect the difference between different measurement points. Since the purpose of this paper is to obtain a better pseudo-color imaging effect without processing the Raman data, the Raman spectral data is measured twice and averaged. Therefore, the maximum value is selected for each measured point, and the grayscale image of size a×b (the unit is a pixel, a represents the length, b represents the width) is arranged according to the position of the observation point, as shown in the following Figure 12. Since there are very few observation points, usually around a few hundred observation points, how to convert them into a visible high-resolution image is a difficulty.

**Figure 13:**
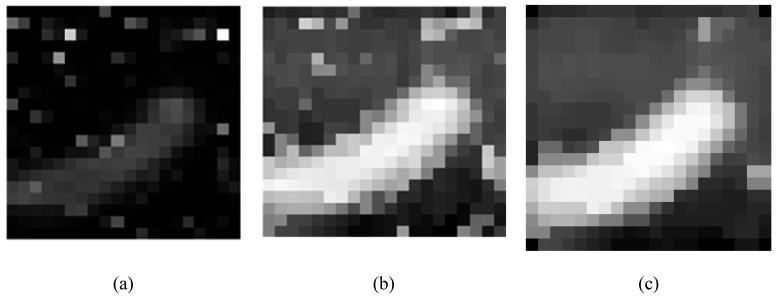
(**a**) Original Raman grayscale image. (**b**) Image processed by the Retinex theory. (**c**) Median filtered image.

After obtaining a grayscale image of a×b size, as shown in the following Figure 13a, the image contrast is too small, and it is difficult to distinguish the objects in the image. Therefore, the Retinex method is used to improve the contrast of the image, as shown in Figure 13b below. The noise in the image is prominent, similar to the salt particle noise. Because the image size is too small, the image is processed using the median filtering method. The processing result is shown in Figure 13c, the cell outline in the image is obvious, and the salt particle noise is better filtered.

**Figure 14:**
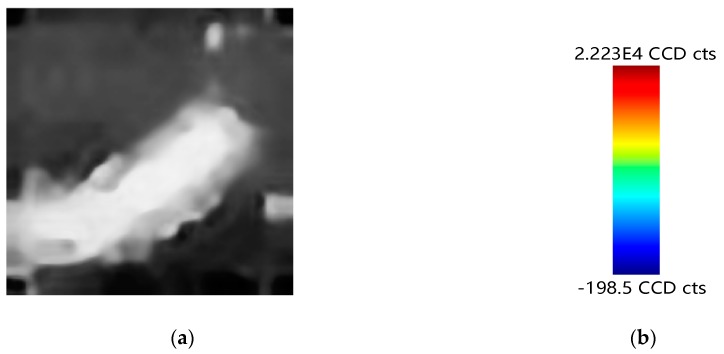
(**a**) The grayscale image after interpolation and super-resolution. (**b**) Pseudo-color index sequence.

The image is processed by super-resolution below. Considering the imaging effect and the algorithm complexity, this paper selects the deep neural network SRCNN network with few layers which has been pre-trained in the ImageNet database. Perform two SRCNN operations, and finally get a visually large grayscale image, as shown in Figure 14a.

Due to the blurry image, the adaptive guided filter is used for image enhancement processing. The parameters |ω| and eps are calculated by the following linear equation $\left[\begin{array}{c} \left|\omega \right|\\ eps \end{array}\right]=f\left(N_{1},N_{2},\sigma_{k}^{2}\right)=\left[\begin{array}{c} 0.0113\\ -0.0797 \end{array}\right]N_{1}+\left[\begin{array}{c} -0.0056\\ 0.1210 \end{array}\right]N_{2}+\left[\begin{array}{c} 0.0001\\ -0.0001 \end{array}\right]\sigma_{k}^{2}+\left[\begin{array}{c} 3.1181\\ 44.2304 \end{array}\right]$. The full-band data is imaged and pseudo-color processed, using the Jet pseudo-color index sequence in MATLAB, as shown in the Figure 14b. The Raman spectral pseudo-color imaging task was finally completed.

**Figure 15:**
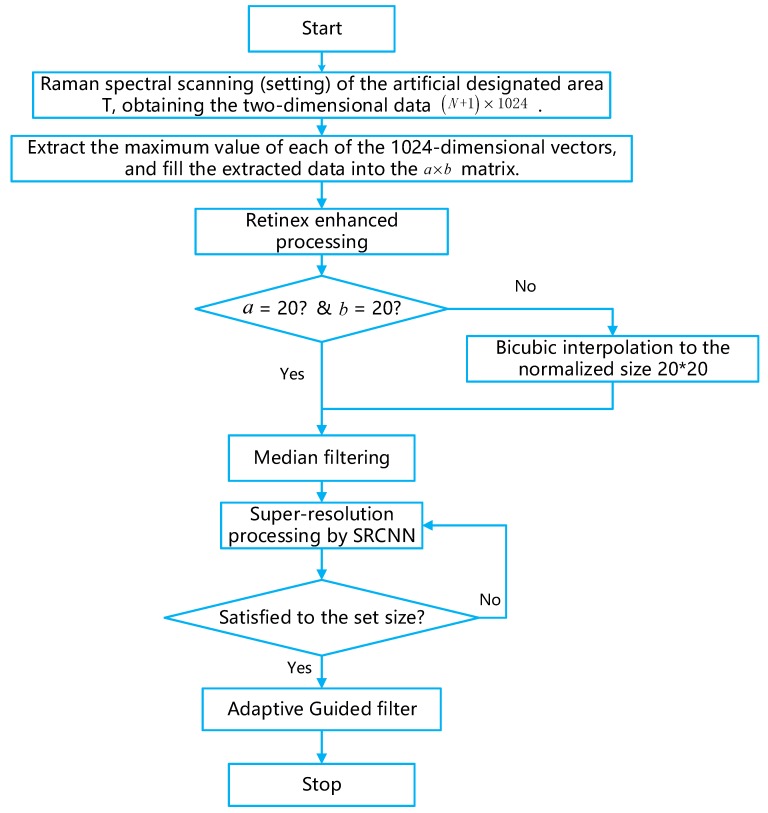
The flow chart of this paper’s algorithm.

The architecture of the paper algorithm is shown in Figure 15 and Algorithm1:

**Algorithm1.** Super-resolution algorithm of cell pseudo-color image based on Raman spectrum.
**Input: Two-dimensional Raman spectroscopy raw data of size (N + 1) × 1024.**
Output: Pseudo-color cell image1. Select the peak information of the bands in the N measurement points and arrange them in a matrix form according to the measurement points.2. Normalize the peak information to obtain a grayscale image.3. Use Retinex enhancement processing on the images.4. If (size_a < 20) and (size_b < 20) 5. Interpolate to at least 20 6. Use median filtering 7. Put the gray image into the SRCNN  If the pixel size < 320    Use SRCNN to realize the image super-resolution. Until the pixel size is equal to the 320. 8. Use Adaptive Guided filter to smooth the image 9. Use the Jet Index Table to realize the super-resolution pseudo-color imaging

## 4. Results and Discussion

**Figure 16:**
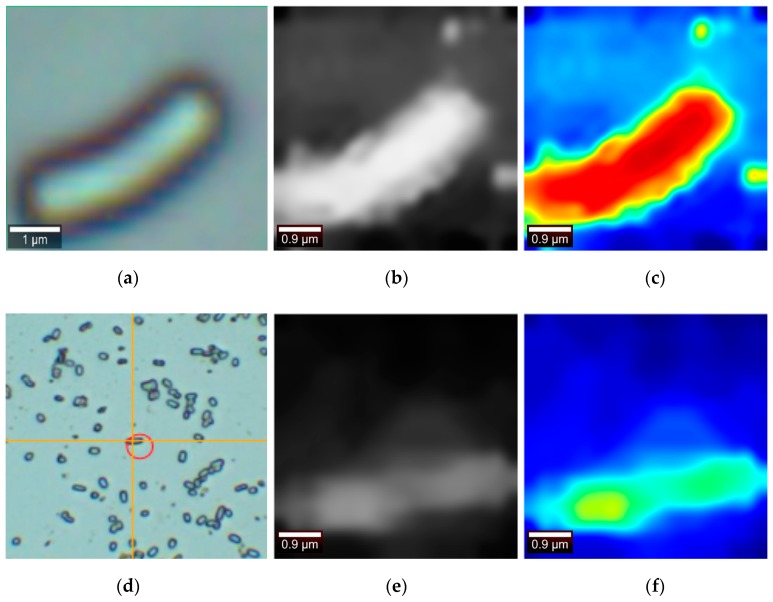
(**a**,**d**) Digital optical microscope image. (**b**,**e**) The Raman grayscale image generated by the algorithm presented by this paper in the full band. (**c**,**f**) Pseudo-color Raman image obtained using the algorithm of the present paper.

**Figure 17:**
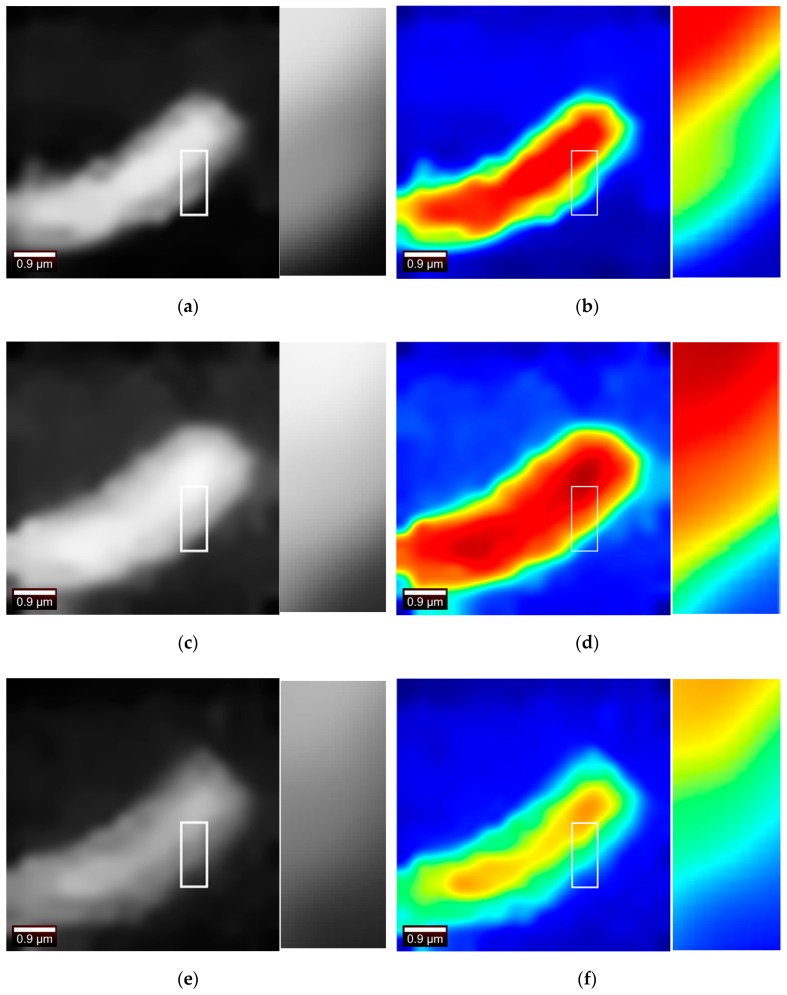
(**a**,**b**) Imaging in the band 50–2750 (cm^−1^) (**c**,**d**) Imaging in the band 2750–3050 (cm^−1^) (**e**,**f**) Imaging in the band 3050–3950 (cm^−1^).

The data of Figure 16b,c below is acquired under the following conditions: A 20 × 20 measurement dot matrix was selected, and a Raman scattering point scan was performed on the Escherichia coli (dh5a strain) at 25 °C to obtain the following image. The integral power is 1.5 mw and the integration time is 2 s. The lens parameter is 600 g/mm and the same scan point is scanned twice. The data of the Figure 17e,f below is under the following conditions: A 10 × 10 measurement point was selected, and a Raman scattering point scan was performed on the Escherichia coli (dh5a strain) at 25 °C. The integral power is 1.5 mw, and the integration time is 2 s. The lens parameter is 600 g/mm, and the same scan point is scanned twice. Since the image size of 10 × 10 is too small, the Retinex enhancement processing is performed on the 10 × 10 images. Then, using the bilinear interpolation processing unifies the image into the size of 20 × 20, and the use of the median filtering to process the noise. Since no algorithm papers related to pseudo-color imaging of Raman cells have been found, this paper mainly discusses two aspects: software vs. software, algorithm vs. algorithm.

### 4.1. Comparison with A Digital Optical Microscope

The full-band data is taken into the method proposed in this paper for calculation, and the results of the following Figure 16b,c,e,f are obtained.

The image under the digital optical microscope is taken as a reference image. By observing the pseudo-color image, the contour of the cell can be clearly observed and is similar to the cell size under an digital optical microscope. The pseudo-color imaging has significant contrast. The disadvantage; however, is that the image contains significant noise and Figure 16e only roughly depicts the cell outline, with less information about the inside of the cell.

### 4.2. Imaging Contrast and Analysis for Different Bands

In view of the Figure 9a Raman waveform diagram, three bands were selected for analysis, which were 50–2750, 2750–3050, 3050–3950 (unit: cm^−1^). Two sets of experimental data were subjected to Raman pseudo-color image processing in three bands.

The first set of data is processed in the bands 50–2750, 2750–3050, 3050–3950 (unit: cm^−1^) to obtain three sets of images, as shown in the following Figure 17.

The peak signal-to-noise ratio (PSNR) is used to evaluate the performance of the algorithm. The peak signal-to-noise ratio is evaluated by the mean square error (MSE) for the similarity of the two images. Two images are define with length a and width b as I and K, respectively, and the mean square error is:

(19) $$M_{\mathrm{SE}}=\frac{1}{a\times b}\overset{a}{\underset{i=1}{\sum }}\overset{b}{\underset{j=1}{\sum }}{\left\|I\left(i,j\right)-K\left(i,j\right)\right\|}^{2}$$

and the calculation equation of the peak signal to noise ratio is:

(20) $$P_{\mathrm{SNR}}=20\cdot \log_{10}\left(\frac{255}{\sqrt{M_{\mathrm{SE}}}}\right)$$

Cell contours could be seen in all three bands. The PSNR values are obtained from each other for the three grayscale images obtained as shown in the following Table 1.

Observing the above table, it is found that the PSNR values of Figure 17a,c are larger, indicating that Figure 17a is closer to Figure 17c. The key information in this spectrum is in the second band; therefore, both the first band and the third band are missing certain information, so the images look more blurred. The effect that the three images could see the cell contour demonstrates from the side that the system is better robust.

**Figure 18:**
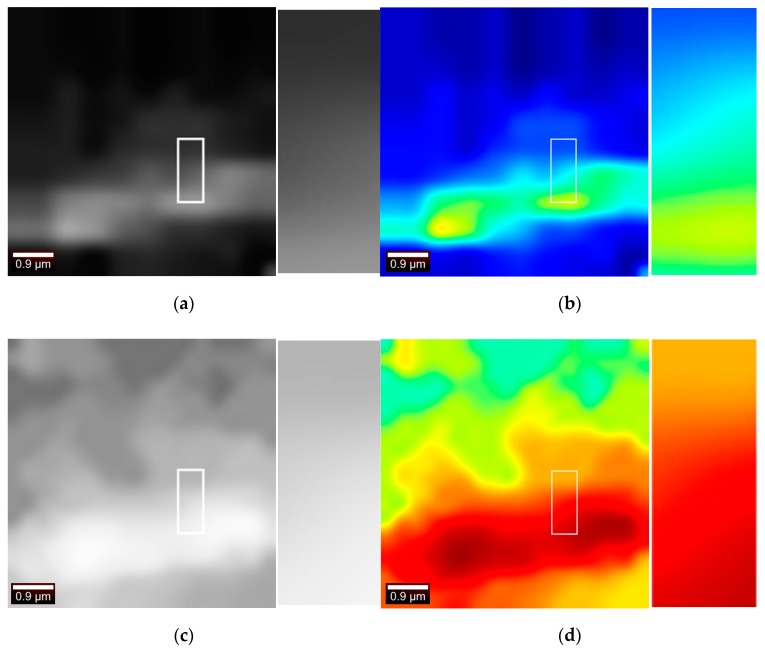

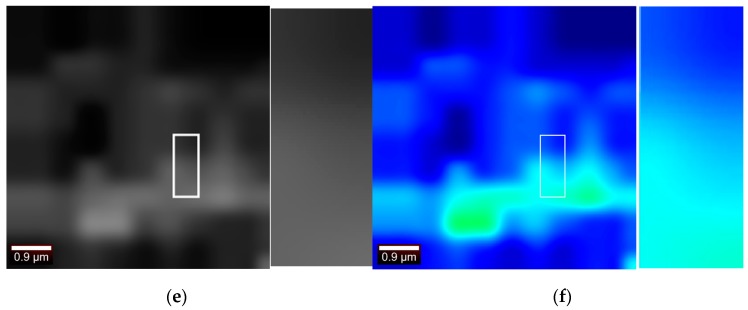
(**a**,**b**) Imaging in the band 50–2750 (cm^−1^) (**c**,**d**) Imaging in the band 2750–3050 (cm^−1^) (**e**,**f**) Imaging in the band 3050–3950 (cm^−1^).

The second set of data is processed in the bands 50–2750, 2750–3050, 3050–3950 (unit: cm^−1^) to obtain three sets of images, as shown in the following Figure 18.

It can be seen that the shape of the cells can be seen by imaging regardless of the band. As shown in the Table 2, the PSNR values are obtained from each other for the three grayscale images.

Observing the above table, similar to the results of previous experiment, it is found that the Figure 18a,e have larger PSNR values, while Figure 18c has lower similarity with the other two images. The reason is that the key information of the spectrum is in the second band. The first band and the third band are missing important information, so the image looks fuzzier and more similar. This experiment is inferior to the previous experiment imaging effect. The reason is that the measurement points are selected less, so the Raman information measured by the image contains more noise. However, the pseudo-color imaging of Band 1 and Band 3 can still see the cell outline and position

### 4.3. Pseudo-color Super-resolution Algorithm Comparison

Since the pseudo-color images obtained by using this paper’s algorithm are images with no reference type, it is difficult to have an evaluation of the superiority of the fair comparison algorithm. In this paper, we use the cell images, which are interpolated and blurred. Compare the different algorithms, such as ‘Yang et al.’ [34], ‘Zeyde et al.’ [35], ‘GR’ [36], ‘ANR’ [36], ‘NE +L E’ [37], ‘NE + NNLS’ [38], ‘A+’ [36], ‘SRCNN’. The evaluation methods about PSNR, SSIM, NQM, GSM, and MSSIM are created on the cell pseudo-color dataset (Built by the laboratory itself). The evaluation results are shown in the Table 3. The original images are images that have not been super-resolution processed. When evaluating the image quality, the larger the difference between the original image and the processed image, the better the super-resolution effect. Therefore, the bold blackbody numbers in the table indicate that the SRCNN super-resolution is superior to other super-resolution algorithms.

### 4.4. Algorithm Sharpness Comparison

Since the images obtained in this paper are no-reference images, refer to the method used in [39]. Convert the image space from RGB to CIELAB, and use the Kullback–Leiber (KL) divergence to quantize the difference between the probability densities of two random variables (i.e., the two images compared). This method could assess the degree of visual clarity through information theory measurement methods.

Suppose and represent the probability mass functions of the two images to be compared in CIELAB space, respectively, and define the comparison formula as

(21) $$D\left(p\|q\right)=\left|\underset{L*,C^{*},h^{*}}{\sum }p\left(L^{*},C^{*},h^{*}\right)\log\frac{p\left(L^{*},C^{*},h^{*}\right)}{q\left(L^{*},C^{*},h^{*}\right)}\right|$$

If $p\left(L^{*},C^{*},h^{*}\right)$ is close to $q\left(L^{*},C^{*},h^{*}\right)$, then D(p‖q) will be close to 0, which means that the visual clarity of the two is relatively close.

Comparing the bold blackbody values in the Table 4, we can conclude that the image clarity of the algorithm is much higher than the image generated by the Witec instrument. The comparison is made by the image of digital optical microscopy because the resolution of the digital optical microscope is far higher than the resolution of the Raman spectrum.

### 4.5. Imaging Comparison of Witec Instruments

**Figure 19:**
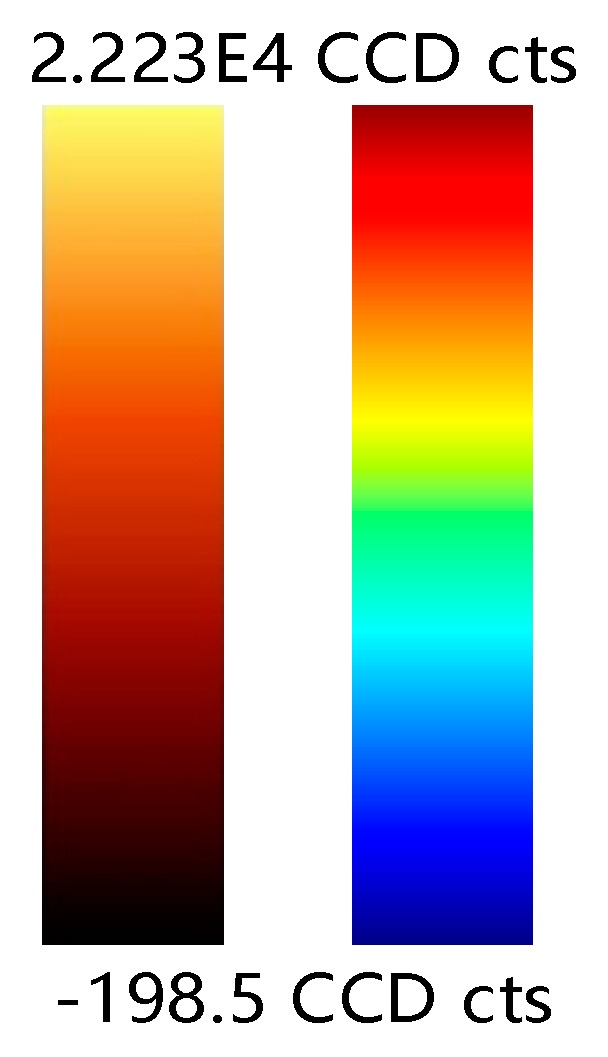
Pseudo-color sequence of Witec instrument (Left), pseudo-color sequence of this paper (Right).

**Figure 20:**
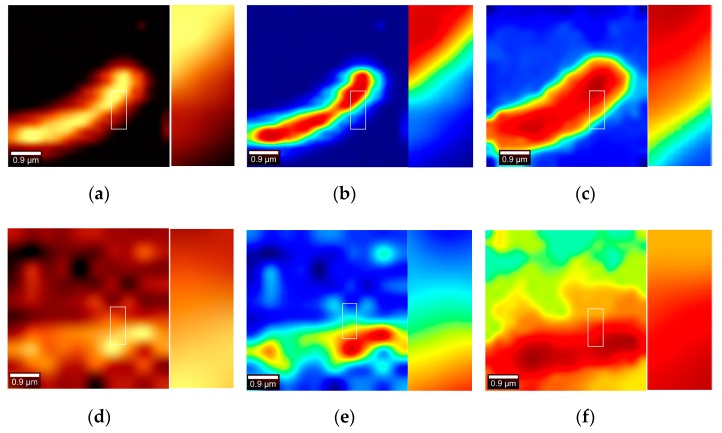
(**a**) Witec pseudo-color imaging in the first set of data in Band 2. (**b**) Witec pseudo-color imaging with the same color bars used in this article. (**c**) Pseudo-color imaging by the method of this paper in the first set of data in Band 2. (**d**) Witec pseudo-color imaging in the second set of data in Band 2. (**e**) Witec pseudo-color imaging with the same color bars used in this article. (**f**) Pseudo-color imaging by the method of this paper in the first set of data in Band 2.

The image of Band 2 imaged by the Witec instrument is compared with the results of the method used in this paper. The pseudo-color sequence selected by Witec instrument and the pseudo-color sequence used in this paper are shown in the following Figure 19:

Witec instrument is used for detection and is divided into 90 observation wavelengths in the wavelength range of 2750–3050. Witec measured the pseudo-color image (a,c) as shown in Figure 20, and the image obtained by this paper’s algorithm were imaged as (b,d). It can be seen that compared with (c) the image is ambiguous and the detailed information cannot be recognized, the algorithm in this paper better highlights the contour of the cell. However, there is still some noise. Compared with the results obtained by Witec, the pseudo-color index sequence used in this paper enhances the contrast of the image and reflects the cell contour. The two experiments indicate that the red region in the image was also verified as a cell.

By analyzing the image information entropy, the amount of information contained in the pseudo-color image is evaluated. The image information entropy equation is:

(22) $$E_{\mathrm{ntropy}}=\overset{255}{\underset{i=0}{\sum }}p_{i}\log p_{i}$$

where $p_{i}=\frac{f\left(i\right)}{\left(a\times b\right)}$, f(i) represents the number of pixels in the image with a statistical gray value of i, where 0≤i≤255 and i∈N. a is the image length and b is the image width.

If the information entropy of the pseudo-color image is calculated, the equation is

(23) $$E_{\mathrm{ntropy}}=\overset{255}{\underset{i,j,k}{\sum }}p_{ijk}\log p_{ijk}$$

where $p_{ijk}=\frac{f\left(i,j,k\right)}{\left(a\times b\times 3\right)}$, the same 0≤i≤255, 0≤j≤255, 0≤k≤255, and i,j,k∈N.

The information entropy of the pseudo-color image generated by the Witec instrument and by this paper is calculated, and the red channel information entropy, the green channel information entropy and the blue channel information entropy of the image are calculated shown in the Table 5, respectively. It is observed from Table 4 that the red channel information entropy and the green channel information entropy of Figure 20a,c generated by the Witec instrument are both high, which may be due to the selected pseudocolor sequence being biased toward black, red, and yellow. Comparing the color image information entropy, it is found that the value obtained in this paper is smaller than that obtained by Witec instrument, which indicates that the method used in this paper is better for cell segmentation of images. Meanwhile, the difference of the entropy value obtained in this paper and by Witec instrument is small, which indicates that the method used in this paper is not over-segmented or under-segmented.

Compared with the Witec instrument imaging using this paper’s color map, the image information entropy or sub-channel information entropy is small, indicating that the image generated by the Witec instrument is relatively smooth. However, from a visual perspective, the resulting boundaries are blurred and the most important point is that the size of the cells has been severely distorted.

## 5. Conclusions

**Figure 21:**
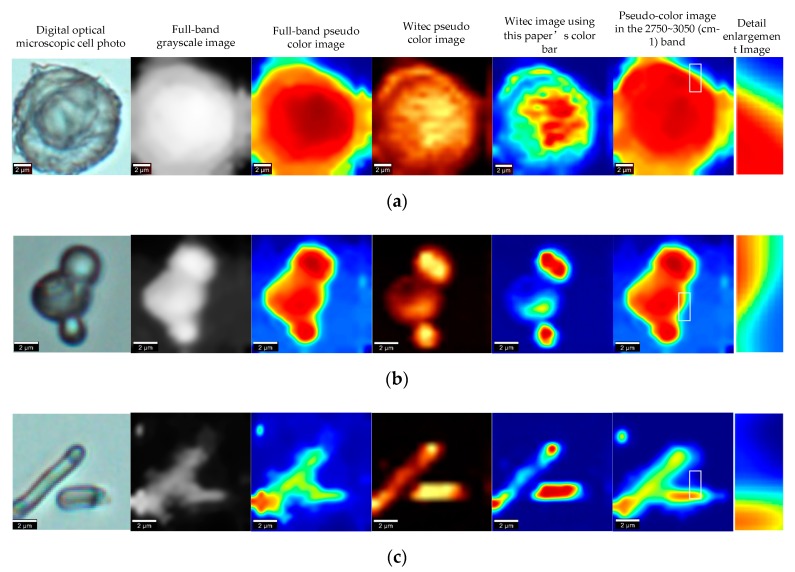
(**a**) Escherichia coli (dh5a strain), integral power is 10.98 mw, integration time is 2 s, integral number is 2, lens parameter is 600 g/mm. (**b**) yeast cells (Yeast), integral power is 1.5 mw, integration time is 5 s, integral number is 2 lens parameter is 600 g/mm. (**c**) human colon cancer cells (hct116 cell line), integral power is 1.5 mw, integration time is 5 s, integral number is 2, lens parameter is 600 g/mm.

In this paper, we propose a novel visualization method for cell Raman spectroscopy, which can be widely used in microscopic cell research. From the perspective of image processing, the peak is extracted without denoising the Raman spectral data, and the super-resolution network is deeply studied. A method of adaptively selecting the radius and penalty coefficient is proposed to generate the cell image. The method has the following advantages: 1) the image is clear, the edge is obvious, and the contour of the photograph under the contrast digital optical microscope is consistent; 2) the universality is obvious, and the image generated by the strong noise or weak noise Raman spectral data is relatively clear. More experimental results are shown in Figure 21, and the corresponding color image entropy and image subchannel entropy are shown in Table 6.

As for future work, there are a few interesting topics that are worth to explore, such as how to detect the substance elements contained in cells, how to perform pseudo-color imaging of Raman spectra of clinical cells which are the problems worth studying.

## Figures and Tables

**Table 1:** The first set of data gray images mutual P_SNR_ values.

Image Serial Number.	a/c	a/e	c/e
*P*_SNR_	16.6071	20.3542	15.5051

**Table 2:** The second set of gray data images mutual P_SNR_ values.

Image Serial Number.	a/c	a/e	c/e
*P*_SNR_	5.5294	22.1526	5.5727

**Table 3:** Comparison of super-resolution results of different algorithms.

	Yang et al.	Zeyde et al.	GR	ANR	NE+LS	NE+NNLS	A+	SRCNN
PSNR(dB)	38.69	38.67	40.35	40.81	38.86	38.41	39.39	**40.0989**
SSIM	0.9949	0.9946	0.9957	0.9954	0.9947	0.9942	0.9951	**0.9886**
NQM	38.4431	40.905	44.2627	43.1203	41.6594	41.4006	43.2622	**18.2565**
GSM	0.9972	0.9993	0.9993	0.9994	0.9994	0.9993	0.9992	**0.9994**
MSSIM	0.9995	0.9995	0.9997	0.9996	0.9995	0.9995	0.9996	**0.9892**

**Table 4:** For the evaluation of image sharpness, it is worth noting that the Third Set, the Fourth Set, the Fifth Set is mentioned at the end of the article.

	Witec// Digital Optical Microscope	Proposed// Digital Optical Microscope	Witec//Proposed
First Set	2.4801	**0.4356**	0.4359
Second Set	3.2007	**0.8530**	0.8525
Third Set	0.8373	**0.1104**	0.1099
Fourth Set	10.1380	**0.9127**	1.0141
Fifth Set	21.6456	**0.2861**	2.2749

**Table 5:** The comparison of image information entropy.

Serial Number	Color Image Information *E*_ntropy_	Red Channel Information *E*_ntropy_	Green Channel Information *E*_ntropy_	Blue Channel Information *E*_ntropy_
(a)	4.3864	5.9122	3.5972	2.4055
(b)	2.8083	1.2747	2.0186	3.5762
(c)	3.7458	2.3701	5.2570	2.5151
(d)	5.9246	7.6206	5.8199	2.2958
(e)	3.8383	2.0761	5.0069	3.5762
(f)	4.8561	4.7647	4.4454	3.5927

**Table 6:** The entropy of the Figure 21 Witec images and images generated by this paper’s algorithm.

Serial Number	Red Channel Information E_ntropy_	Green Channel Information E_ntropy_	Blue Channel Information E_ntropy_	Color Image Information E_ntropy_	Witec Imaging Information E_ntropy_	Witec Imaging Information E_ntropy_ Using This Paper’s Color Bar
(a)	3.4060	4.3614	1.9876	3.6124	6.7437	3.9554
(b)	1.9672	4.9585	2.6200	3.6093	4.7354	2.9855
(c)	1.6811	2.4665	3.8548	3.1869	4.3815	2.6852

## References

[1] Xue L.I., Gao G.M., Niu L.Y., Lin M.M., Qin Z.D., Liu J.X., Yao H.L. (2012). In vivo Raman Imaging of Mice Ear. Chin. J. Anal. Chem..

[2] Fan X.G., Wang X.F., Wang X., Xu Y.J., Que J., Wang X.D., He H., Li W., Zuo Y. (2016). Research of the Raman Signal De-Noising Method Based on Feature Extraction. Guang Pu.

[3] Chen Z., Peng Y., Li Y., Zhao J. (2017). Detection of Chemical Additives in Food Using Raman Chemical Imaging System. Chem. J. Chin. Univ..

[4] Firkala T., Farkas A., Vajna B., Nagy Z.K., Pokol G., Marosi G., Szilágyi I.M. (2015). Quantification of low drug concentration in model formulations with multivariate analysis using surface enhanced Raman chemical imaging. J. Pharm. Biomed. Anal..

[5] Bruckner M., Becker K., Popp J., Frosch T. (2015). Fiber array based hyperspectral Raman imaging for chemical selective analysis of malaria-infected red blood cells. Anal. Chim. Acta.

[6] Schmid T., Dariz P. (2016). Chemical imaging of historical mortars by Raman microscopy. Constr. Build. Mater..

[7] Zhang J., Ma X., Xu M., Zong C., Ren B. (2016). Study on Apoptosis Process of CaSki via Fast Line. scanning Raman Imaging. Chem. J. Chin. Univ..

[8] Bi Y., Yang C., Chen Y., Yan S., Yang G., Wu Y., Zhang G., Wang P. (2018). Light Science Applications. Near-resonance enhanced label-free stimulated Raman scattering microscopy with spatial resolution near 130 nm. Light Sci. Appl..

[9] Ding K., Ning C.Z. (2012). Light Science Applications. Metallic subwavelength-cavity semiconductor nanolasers. Light Sci. Appl..

[10] Lombardini A., Mytskaniuk V., Sivankutty S., Andresen E.R., Chen X., Wenger J., Fabert M., Joly N., Louradour F., Kudlinski A. (2018). High-resolution multimodal flexible coherent Raman endoscope. Light Sci. Appl..

[11] Song J., Zhang X., Yao Z., Hu C., Rui Z., Yang Z., Yuan L., Smith Z.J., Dong Z., Hou J.G. (2017). Subnanometer-resolved chemical imaging via multivariate analysis of tip-enhanced Raman maps. Light Sci. Appl..

[12] Li Q., Zhang J., Shi D., Liu Q. (2016). Determination of Sudan I in duck feed by microscopic image processing and confocal Raman spectroscopy. Anal. Methods.

[13] Chao K., Dhakal S., Qin J., Kim M., Peng Y. (2018). A 1064 nm Dispersive Raman Spectral Imaging System for Food Safety and Quality Evaluation. Appl. Sci..

[14] Yaseen T., Sun D.-W., Cheng J.-H. (2017). Raman imaging for food quality and safety evaluation: Fundamentals and applications. Trends Food Sci. Technol..

[15] Anna I., Bartosz P., Lech P., Halina A. (2017). Novel strategies of Raman imaging for brain tumor research. Oncotarget.

[16] Lohumi S., Lee H., Kim M., Qin J., Cho B.K. (2018). Raman Imaging for the Detection of Adulterants in Paprika Power: A Comparison of Data Analysis Methods. Appl. Sci..

[17] Hauke K., Kehren J., Böhme N., Zimmer S., Geisler T. (2019). In Situ Hyperspectral Raman Imaging: A New Method to Investigate Sintering Processes of Ceramic Material at High-temperature. Appl. Sci..

[18] Wada Y., Enokida I., Yamamoto J., Furukawa Y. (2018). Raman imaging of carrier distribution in the channel of an ionic liquid-gated transistor fabricated with regioregular poly(3-hexylthiophene). Spectrochim. Acta Part A Mol. Biomol. Spectrosc..

[19] Kopeć M., Abramczyk H. (2018). Angiogenesis—A crucial step in breast cancer growth, progression and dissemination by Raman imaging. Spectrochim. Acta Part A Mol. Biomol. Spectrosc..

[20] Bilo F., Zanoletti A., Borgese L., Depero L.E., Bontempi E. (2019). Chemical Analysis of Air Particulate Matter Trapped by a Porous Material, Synthesized from Silica Fume and Sodium Alginate. J. Nanomater..

[21] Cheng J.X., Xie X.S. (2015). Vibrational spectroscopic imaging of living systems: An emerging platform for biology and medicine. Science.

[22] Shinzawa H., Awa K., Kanematsu W., Ozaki Y. (2010). Multivariate data analysis for Raman spectroscopic imaging. J. Raman Spectrosc..

[23] Nascimento J.M.P., Dias J.M.B. (2005). Vertex component analysis: A fast algorithm to unmix hyperspectral data. IEEE Trans. Geosci. Remote Sens..

[24] Ando J., Palonpon A.F., Sodeoka M., Fujita K. (2016). High-speed Raman imaging of cellular processes. Curr. Opin. Chem. Biol..

[25] Qin J., Kim M.S., Chao K., Schmidt W.F., Cho B.K., Delwiche S.R. (2016). Line-scan Raman imaging and spectroscopy platform for surface and subsurface evaluation of food safety and quality. J. Food Eng..

[26] Land E.H., Mccann J.J. (1971). Lightness and retinex theory. J. Opt. Soc. Am..

[27] Jobson D.J., Rahman Z., Woodell G.A. (1997). Properties and performance of a center/surround retinex. IEEE Trans. Image Process..

[28] Dong C., Loy C.C., He K., Tang X. (2016). Image Super-Resolution Using Deep Convolutional Networks. IEEE Trans. Pattern Anal. Mach. Intell..

[29] Farbman Z., Fattal R., Lischinski D. (2008). Edge-preserving decompositions for multi-scale tone and detail manipulation. ACM Trans. Graph..

[30] Yang Q., Tan K.H., Ahuja N. In Real-time O(1) bilateral filtering. Proceedings of the IEEE Conference on Computer Vision and Pattern Recognition.

[31] He K., Jian S., Tang X. (2010). Guided Image Filtering, European Conference on Computer Vision 2010.

[32] Gastal E.S.L., Oliveira M.M. (2011). Domain transform for edge-aware image and video processing. ACM Trans. Graph..

[33] Han Q.Y., Zhou P.J. (2015). Research on the Method of Eliminating Noise and Background in the Meantime in Detecting Ethanol Contention Based on Raman Spectra. Guang pu.

[34] Yang J., Wright J., Huang T.S., Ma Y. (2010). Image Super-Resolution via Sparse Representation. IEEE Trans. Image Process..

[35] Zeyde R., Elad M., Protter M. (2012). On Single Image Scale-Up Using Sparse-Representations. International Conference on Curves & Surfaces.

[36] Timofte R., De V., Gool L.V. Anchored Neighborhood Regression for Fast Example-Based Super-Resolution. Proceedings of the IEEE International Conference on Computer Vision.

[37] Chang H., Yeung D.Y., Xiong Y. Super-resolution through neighbor embedding. Proceedings of the IEEE Computer Society Conference on Computer Vision & Pattern Recognition.

[38] Pan L., Peng G., Yan W., Zheng H. (2016). Single image super resolution based on multiscale local similarity and neighbor embedding. Neurocomputing.

[39] Fiset P.O., Soussi-Gounni A., Christodoulopoulos P., Tulic M., Sobol S.E., Frenkiel S., Lavigne F., Lamkhioued B., Hamid Q. A measure for evaluation of the information content in color images. Proceedings of the IEEE International Conference on Image Processing.

